# Cytology of Primary Salivary Gland-Type Tumors of the Lower Respiratory Tract: Report of 15 Cases and Review of the Literature

**DOI:** 10.3389/fmed.2017.00043

**Published:** 2017-04-24

**Authors:** Chiara Saglietti, Marco Volante, Stefano La Rosa, Igor Letovanec, Marc Pusztaszeri, Gaia Gatti, Massimo Bongiovanni

**Affiliations:** ^1^Service of Clinical Pathology, Institute of Pathology, Lausanne University Hospital, Lausanne, Switzerland; ^2^Department of Oncology, University of Turin at San Luigi Hospital, Turin, Italy; ^3^Department of Pathology, Geneva University Hospital, Geneva, Switzerland

**Keywords:** cytology, lung, salivary gland-type tumors, mucoepidermoid carcinoma, adenoid cystic carcinoma

## Abstract

Primary pulmonary salivary gland-type tumors are rare neoplasms arising from the seromucinous submucosal glands of the lower respiratory tract (LRT), the most common of which are mucoepidermoid carcinoma (MEC) and adenoid cystic carcinoma. They are morphologically indistinguishable from their salivary gland counterpart and recognizing them is a challenge, especially on cytological specimens. We analyzed 15 cases of histologically proven primary salivary gland tumors of the LRT to identify cytomorphological features and define potential diagnostic clues that might assist cytopathologists in the preoperative diagnosis of these neoplasias. Three out of the four cases of adenoid cystic carcinomas showed the characteristic tridimensional cell clusters and hyaline globules, whereas the last one did not show malignant cells; only two cases of MEC presented the three characteristic cell types (i.e., squamous, intermediate, and mucin secreting) on cytology. Since these neoplasms are rare and do not have a completely specific set of cytological features, it is important for practicing cytopathologists to be aware of the possibility of encountering them, in specimens from patients with LRT masses, in order to render the correct diagnosis.

## Introduction

Primary salivary gland-type tumors (PSGT) arising from the seromucinous submucosal glands of the lower respiratory tract (LRT) (which includes trachea, bronchus and lung) account for <1% of central airway carcinomas (1). They are rare neoplasms morphologically indistinguishable from their salivary gland counterpart; therefore, recognizing them is a challenge, especially on cytology. Even though any type of salivary gland tumor that has been described in pathology textbooks can potentially arise in the LRT, published data show that the most commonly encountered primary salivary gland-type tumors in this anatomical site are malignant mucoepidermoid carcinoma (MEC), adenoid cystic carcinoma (AdCC), and epithelial-myoepithelial carcinoma (2–5). In fact, as opposed to the head and neck region, where the vast majority of salivary gland primaries are benign—with pleomorphic adenoma (PA) being the most common type—the contrary applies to LRT primaries (1).

Cytological examination of fine-needle aspiration (FNA), bronchial aspiration (BA) or brushing (BB), bronchoalveolar lavage (BAL), or even sputum has been shown to be a powerful tool for the diagnosis of lung cancer, particularly when it presents as an endobronchial growth. Moreover, in recent years, endobronchial ultrasound-guided transbronchial needle aspiration (EBUS-TBNA) has emerged as the standard of care for the diagnosis and staging of lung cancer and has been successfully implemented into daily clinical practice. EBUS-TBNA is minimally invasive, safe, cost-effective, and particularly useful in diagnosing centrally located lung lesions (6).

All the aforementioned cytological procedures are useful for collecting material for cytological examination, immunocytochemistry (ICC), fluorescent *in situ* hybridization (FISH), or molecular analyses, which may be relevant for diagnosis and targeted therapy. Moreover, in 30% of cases, cytological material is the only material available for pulmonary malignancies and proper classification of the lesions, often with subtyping, is fundamental for adequate patient management.

We describe 15 cases of histologically proven PSGT of the LRT; all but two were misdiagnosed on preoperative cytology. We have tried to identify cytomorphological features that could point to a correct cytological diagnosis. To the best of our knowledge, this is the largest cytological series of PSGT of the LRT ever published.

## Materials and Methods

### Case Selection

Nineteen cases of surgically resected PSGT originating from the trachea, main bronchi, and lung with corresponding preoperative cytology were identified by searching the databases of our institutions (Service of Clinical Pathology, Lausanne University Hospital; Department of Pathology, Geneva University Hospital; Section of Anatomic Pathology, San Luigi Hospital, Orbassano, Turin) over a period of 21 years (1995–2015). Cytological and histological specimens of each case were retrieved from the archives to be reviewed for adequacy. Three cases were excluded because slides were no longer available for revision, and one case was excluded because it originated from the larynx and not from the LRT. The database was also investigated to exclude the presence of primary salivary gland tumors that could have metastasized to the LRT.

The study cohort of the present work was thus composed of 15 cases. Clinical, radiological, and pathological reports for each patient were analyzed to collect pertinent information, including age, gender, alcohol and smoking history, presenting symptoms and signs, radiological findings, tumor size, and original preoperative cytological diagnosis.

### Cytomorphological Features

All cytological smears were reviewed by an expert cytopathologist (Massimo Bongiovanni) to evaluate the presence of cytomorphological features that could have pointed to a correct preoperative diagnosis, namely: the presence of mucin and the three different neoplastic cellular components (mucin secreting, squamous, and intermediate) characteristic of MEC (3); organoid cell clusters, hyaline globules, cellular uniformity, and granular cytoplasm distinguishing AdCC (7). Particular attention was paid to look either cytologically or histologically for some of the newly described entities of salivary gland tumors, namely the mammary analog secretory carcinoma (MASC), the cribriform adenocarcinoma of the tongue, and minor salivary gland (CATS) that so far have never been described in the LRT (8).

## Results

### Clinicopathological Findings

A summary of all relevant clinical, radiological, and pathological data of the patients are presented in Table 1. Patients ranged in age from 16 to 87 years (mean 59.6 ± 18.6 years); there were nine males and six females. From histology, 11 cases were diagnosed as MEC (5 low grade and 6 high grade), and the remaining four cases were diagnosed as AdCC according to the histological criteria defined by the current WHO classification (3, 4).

**Table 1:** **Clinicopathological and radiological data of our patients**.

No.	Sex	Age	Alcohol/smoking	Relevant clinical findings	Radiology/bronchoscopy findings	Site	Lesion size (cm)	Preoperative cytology			Histologic diagnosis	Revised cytological diagnosis
								*BA*	*BB*	*BAL*		
1	F	64	NA/NA	NA	Distal carinal stenosis	Carina	2.5	Salivary gland-type neoplasia	NP	NP	AdCC	AdCC

2	F	74	NA/NA	History of breast ductal carcinoma	Bronchial polypoid mass	Right main bronchus	4.5	Metastatic breast carcinoma		NP	AdCC	AdCC

3	M	70	NA/no	NA	Lung mass	Right superior lobe	1.7	Absence of malignant cells	NP	NP	AdCC	Absence of malignant cells

4	F	75	NA/NA	Weakness, non-productive cough	NA	NA	NA (bioptic material only)	Suspicious for carcinoma	NP	NP	AdCC	AdCC

5	M	87	No/no	Fall with costal fracture, hemorrhagic pleural effusion	Mass lesion with bronchial stenosis and atelectasis	Right lung	2.0	PDC		NP	MEC (low-grade)	PDC

6	F	49	Yes/yes	Weight loss, dyspnea, retrosternal pain	Lung mass	Left upper lobe	3.5	Atypical squamous cells	NP	NP	MEC (low-grade)	PDC

7	F	65	No/no	Weakness, productive cough, hemoptysis	Parahilar mass with atelectasis	Left upper lobe	2.6	PDC	NP	NP	MEC (high-grade)	PDC

8	M	75	No/yes	Progressive dyspnea, non-productive cough	Bronchial stenosis	Left main bronchus	4.0	Suspicious for carcinoma	NP	NP	MEC (high-grade)	PDC

9	M	60	Yes/yes	Ongoing cough	Peribronchial mass lesion	Left inferior lobe bronchus	5.0	NP	Adenocarcinoma	NP	MEC (high-grade)	PDC

10	M	57	NA/NA	NA	Lung nodule	Medium lobe	2.0	Absence of malignant cells			MEC (low-grade)	Adenocarcinoma (for the BA specimen only)

11	M	35	NA/NA	NA	Extrinsic bronchial compression	Apical bronchus of right superior lobe	5.0	NP	Absence of malignant cells		MEC (high-grade)	Absence of malignant cells

12	M	37	NA/NA	NA	Lung mass	Segmental bronchus of right superior lobe	2.0	NP	NSCLC, compatible with MEC		MEC (high-grade)	NSCLC, compatible with MEC

13	M	76	NA/yes	NA	Apical nodule hypermetabolic at PET scan	Left inferior lobe	2.5	Absence of malignant cells			MEC (high-grade)	Absence of malignant cells

14	M	16	No/no	Progressive dyspnea, cough	NA	NA	NA (bioptic material only)	Absence of malignant cells	NP	NP	MEC (low-grade)	Absence of malignant cells

15	F	54	NA/NA	Pleural effusion	NA	NA	NA (bioptic material only)	Suspicious for carcinoma, NOS	NP	NP	MEC (low-grade)	NSCLC, compatible with MEC

*AdCC, adenoid cystic carcinoma; MEC, mucoepidermoid carcinoma; BA, bronchial aspiration; BB, bronchial brushing; BAL, bronchoalveolar lavage; PDC, poorly differentiated carcinoma; NSCLC, non-small cell lung cancer; NA, not available; NP, not performed*.

The cytological slides that were revised included: 12 BA, 7 BB, 5 BAL, and 1 FNA. More than one type of cytological sample was available for 6 out of the 15 cases (Table 1). The smears were either alcohol-fixed, Papanicolaou (PAP) stained or air dried, May-Grünwald-Giemsa (MGG) stained. Neither FISH analysis nor molecular studies were originally performed.

Tumors were all centrally located and ranged in size from 1.7 to 5.0 cm (mean 4.4 ± 1.2 cm). Only one AdCC and one MEC were somehow identified preoperatively: the AdCC was diagnosed as a salivary gland-type neoplasia and the MEC as a non-small cell lung carcinoma, consistent with MEC. Five preoperative cytological cases were originally reported as negative for malignant cells (33.3%) (1 AdCC and 4 MEC), and this diagnosis was confirmed after revision of the slides in four out of five cases. Revised cytological diagnosis of the fifth case was that of an adenocarcinoma (concerning the BA specimen only). Interestingly, one AdCC was misdiagnosed as a metastatic breast carcinoma (due to the previous history of ductal breast carcinoma in the patient). During revision of the slides, all three diagnostic cases of AdCC showed the characteristic tridimensional cell clusters and hyaline globules that permit the cytological diagnosis of this entity, whereas only two cases of MEC presented the three characteristic cell types (i.e., squamous, intermediate, and mucin secreting) on cytology.

## Discussion

Cytology has proven to be a powerful tool for the diagnosis of primary lung cancer. A summary of all published cases of PSGT of the LRT for which a cytological diagnosis is available in the literature is provided in Table 2. Exfoliative cytology, in particular bronchial brushing, aspiration, and washing, is especially useful for tumors with endobronchial growth. PSGT of the LRT, because of their origin from the submucosal bronchial glands, mainly present as endobronchial masses (1), and therefore, they are considered as accessible for cytological sampling and diagnosis. However, as previously reported by other authors, primary pulmonary AdCCs and MECs are usually covered by intact respiratory epithelium; therefore, FNA may be more effective than exfoliative cytology in diagnosis for some of such cases (9, 10). The results from our study confirm that when using exfoliative cytology only, a significant proportion of PSGT of the LRT cases (33%) do not yield diagnostic tumor cells.

**Table 2:** **Summary of all reported cases of primary salivary gland-type tumors of the lower respiratory tract for which cytological diagnosis is available in the literature**.^a^

Reference	Sex	Age	Presentation	Radiology findings	Bronchoscopy findings	Site	Lesion size (cm)	Preoperative cytology					Frozen section	Histologic diagnosis
								*FNA (TT)*	*FNA (TM)*	*BB*	*BW/TW*	*Sputum*		
Tao and Robertson (9) pt no. 1	F	46	Cough, shortness of breath, decreased energy	Well-circumscribed, round lesion (CT)	Mass occluding the right upper lobe bronchus	Right hilum	3	MEC	NA	NA	NA	Negative for malignancy	NA	MEC
Tao and Robertson (9) pt no. 2	F	54	Incidental finding on chest X-ray	Coin lesion (chest X-ray)	NA	Right upper lobe	NA	MEC	NA	NA	NA	NA	NA	MEC (low-grade)
Lozowski et al. (10) pt no. 1	F	40	Productive cough, fever, chills, headache, lethargy	Consolidative pneumonitis of left lower lobe	Polypoid friable tumor	Left main stem bronchus, carinal level	NA	NA	NS	Negative for malignancy	AdCC	AdCC	AdCC	AdCC
Nguyen (11) pt no. 1	M	50	Cough, hemoptysis	NA	NA	Tracheal carina + stem bronchi	NA	NA	NA	NA	NA	AdCC	NA	AdCC
Nguyen (11) pt no. 2	F	36	Cough, hemoptysis	NA	NA	Left stem bronchus	NA	NA	AdCC	AdCC	NA	Positive for malignancy	NA	AdCC
Nguyen (11) pt no. 3	M	48	Persistent cough	NA	NA	Tracheal carina + stem bronchi	NA	NA	MEC (low-grade)	Negative for malignancy	NA	Negative for malignancy	NA	MEC (low-grade)
Nguyen (11) pt no. 4	F	29	Persistent cough	NA	NA	Left stem bronchus	NA	NA	MEC (low-grade)	Negative for malignancy	NA	Negative for malignancy	NA	MEC (low-grade)
Nguyen (11) pt no. 5	M	80	Cough, hemoptysis, weight loss	NA	NA	Right upper lobe bronchus	NA	Adeno-squamous carcinoma	NA	Positive for malignancy	NA	Negative for malignancy	NA	MEC (high-grade)
Buchanan et al. (12) pt no. 1	M	23	Substernal discomfort, choking sensation, wheezing, productive cough	Normal chest X-ray	Obstructing tumor	Trachea	NA	NA	NA	NA	AdCC	NA	NA	AdCC
Buchanan et al. (12) pt no. 2	F	51	Cough, wheezing, intermittent breathing difficulties	Spherical mass	NA	Trachea	1	NA	NA	NA	AdCC	Negative for malignancy	NA	AdCC
Gupta and McHutchison (13) pt no. 1	F	85	Increasing shortness of breath, productive cough	NA	Endotracheal tumor	Midtrachea	NA	NA	NA	NA	AdCC	NA	NA	AdCC
Brooks and Baandrup (14) pt no. 1	M	66	Incidental finding on chest X-ray	Peripheral lung mass	NA	Right lower lobe	4	NA	NA	Negative for malignancy	NA	NA	NA	MEC
Radhika et al. (15) pt no. 1	M	45	Progressive breathlessness, productive cough	Collapse of the right lung	Tumor at the carina extending in the bronchi	Carina + adjacent stem bronchi	NA	NA	NA	NA	AdCC	NA	NA	AdCC
Segletes et al. (16) pt no. 1	M	47	Chronic pneumonia, increasing cough	Central right upper lobe mass	NA	Right upper lobe	NA	MEC	NA	NA	NA	NA	NA	MEC
Segletes et al. (16) pt no. 2	M	72	Incidental finding on chest X-ray	Left lung mass extending into the chest wall	NA	Left lung	NA	Consistent with MEC	NA	NA	NA	NA	NA	MEC
Segletes et al. (16) pt no. 3	M	16	Pneumonia, cough, earache, weight loss	Mediastinal mass with enlarged lymph nodes	NA	Right main stem bronchus	4	NA	NA	NA	NA	NA	NA	MEC
Segletes et al. (16) pt no. 4	F	25	NA	NA	Tumor in the bronchial lumen		NA	NA	NA	AdCC		NA	NA	AdCC
Delpiano et al. (17) pt no. 1	M	52	Cough, hemoptysis	Coin lesion upper lobe of left lung	Reddish cauliflower-like lesion	Upper left lobe bronchus	NA	NA	NA	Papillary structures lined by cuboidal-to-columnar cells with mucin-rich cytoplasm	NA	NA	NA	Papillary mucous gland adenoma
Romagosa et al. (18) pt no. 1	F	33	Cough, fever, mucopurulent expectoration, shortness of breath	NA	Intrabronchial polypoid mass	Left main bronchus	NA	NA	Cells with bland nuclei, wide cytoplasm, and intranuclear inclusions; minor population of mucus-secreting cells	NA	NA	Negative for malignancy	NA	MEC (low-grade)
Romagosa et al. (18) pt no. 2	F	39	Incidental finding on chest X-ray	Right lower lobe mass	NA	Right lower lobe	NA	Cells with bland nuclei, wide cytoplasm, and intranuclear inclusions; minor population of mucus-secreting cells	NA	NA	NA	Negative for malignancy	NA	MEC (low-grade)
Qiu et al. (19) pt no. 1	M	51	Left chest and shoulder pain, fever, leg swelling	Atelectasis of left upper lobe	Endobronchial mass	Left upper lobe bronchus	1	NA	AdCC	NA	NA	NA	NA	AdCC
Florentine et al. (20) pt no. 1	F	85	NA	NA	Obstructing tumor	Left main bronchus	NA	NA	NA	NA	Carcinoid tumor or AdCC	NA	NA	AdCC
Chuah et al. (21) pt no. 1	M	44	Throat irritation, persistent cough	Mass lesion	Polypoid tumor in bronchial lumen	Left hilum	NA	NA	NA	NA	Carcinoma consistent with AdCC	NA	NA	AdCC
Daneshbod et al. (22) pt no. 1	F	55	Increasing shortness of breath, productive cough	Mass lesion	NA	Left lower lobe	NA	NA	NA	?	?	NA	NA	AdCC
Daneshbod et al. (22) pt no. 2		65	Progressive breathlessness, productive cough	Collapse of the right lung	Carinal tumor extending in major bronchi	Carina + adjacent stem bronchi	NA	NA	NA	NA	?	Negative for malignancy	NA	AdCC
Özkara and Turan (23) pt no. 1	M	54	Cough, expectoration, hemoptysis, chest pain, and weight loss	Opacity of left upper lobe (X-ray)	Shiny, sessile, polypoid mass	Left mainstem bronchus	4	NA	AdCC, other than classical type	NA	NA	NA	NA	AdCC, solid variant
				Endobronchial mass lesion (CT)										
Chon et al. (24) pt no. 1	F	46	Incidental finding on chest X-ray	Right upper lung mass	NA	Right upper lobe	NA	AdCC	NA	NA	NA	Negative for malignancy	NA	AdCC
Dyhdalo and Chen (25) pt no. 1	F	45	Productive cough	Well-circumscribed nodule (CT)	NA	Right lower lobe bronchus	NA	NA	Low-grade epithelial neoplasm, favor a low-grade bronchial MEC	NA	NA	NA	NA	MEC (low-grade)
Kim et al. (7) pt no. 1	M	42	NA	Bronchial narrowing	NA	Lymph node 1R	NA	NA	NA	NA	Metastatic carcinoma from trachea	NA	NA	AdCC
Kim et al. (7) pt no. 2	F	47	NA	Endobronchial tumor infiltration	NA	Left main bronchus	NA	NA	NA	NA	Positive for malignant cells	NA	NA	AdCC
Kim et al. (7) pt no. 3	M	52	NA	Bronchial obstruction	NA	Lymph node, 7	NA	NA	Metastatic AdCC from lung	NA	NA	NA	NA	AdCC
Kim et al. (7) pt no. 4	F	61	NA	NA	NA	Trachea	NA	NA	AdCC cannot be excluded	NA	NA	NA	NA	AdCC
Kim et al. (7) pt no. 5	M	57	NA	Bronchial obstructing mass	NA	Right lower bronchus	NA	NA	NA	NA	A nest of atypical cells	NA	NA	AdCC
Kim et al. (7) pt no. 6	M	65	NA	Tracheal obstruction	NA	Carina	NA	NA	NA	NA	Atypical cells	NA	NA	AdCC
Kim et al. (7) pt no. 7	F	75	NA	Bronchial narrowing	NA	Left main bronchus	NA	NA	NA	NA	Suspicious for malignancy	NA	NA	AdCC
Kim et al. (7) pt no. 8	M	60	NA	Bronchial obstruction	NA	Right upper bronchus	NA	NA	NA	NA	Suspicious for malignancy	NA	NA	AdCC
Kim et al. (7) pt no. 9	M	53	NA	Tracheal mass	NA	Trachea	NA	NA	NA	NA	AdCC cannot be excluded	NA	NA	AdCC
Kim et al. (7) pt no. 10	F	58	NA	Bronchial obstructing mass	NA	Right main bronchus	NA	NA	NA	NA	Positive for malignant cells	NA	NA	AdCC
Kim et al. (7) pt no. 11	F	55	NA	NA	NA	Trachea	NA	NA	AdCC versus EMC	NA	NA	NA	NA	AdCC
Bhalara et al. (26) pt no. 1	F	20	Exertional dyspnea, dry cough, fever, hemoptysis	Mixed echogenic lesion (US)	NA	Left upper lung	9	AdCC	NA	NA	NA	NA	NA	AdCC

*pt, patient; MEC, mucoepidermoid carcinoma; AdCC, adenoid cystic carcinoma; EMC, epithelial-myoepithelial carcinoma; FNA, fine-needle aspiration; TT, transthoracic; TM, endoscopic transmucosal; BB, bronchial brushing; BW, bronchial washing; TW, tracheal washing; NA, non-available; US, ultrasound*.

*^a^An additional article about primary salivary gland-type tumors of the lower respiratory tract (LRT) exists and includes a series of 18 cases of AdCC (of which 5 arising in the LRT), which were analyzed for a panel of 17 items (27)*.

**Figure 1:**
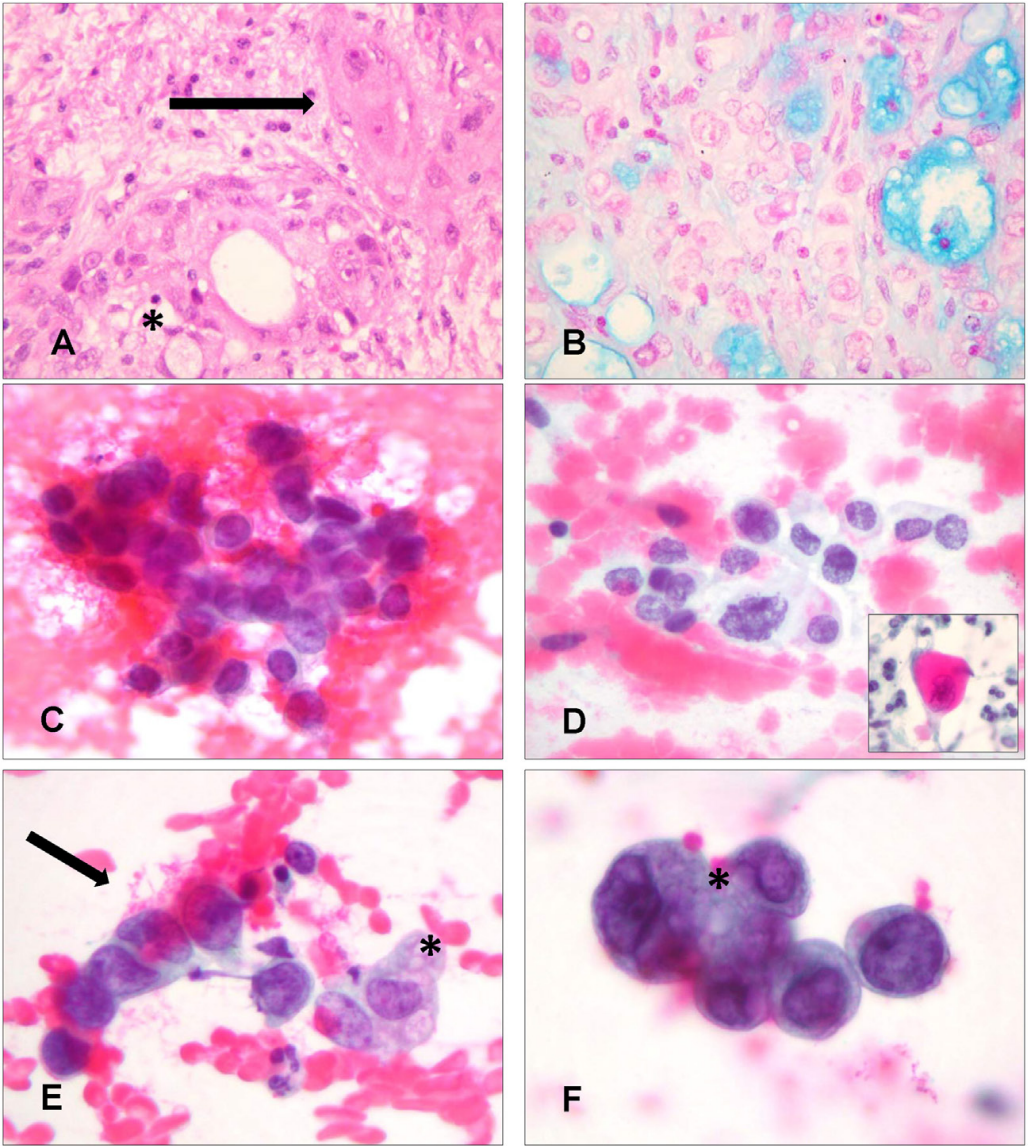
**Histological and cytological aspects of primary mucoepidermoid carcinoma (MEC) of the lower respiratory tract**. **(A)** Histologically, in case 9, diagnosed as a high-grade MEC, both squamous cells (arrow) and mucin-secreting cells (asterisk) are visible (Hematoxylin and Eosin, 400×). **(B)** Mucin-secreting cells are highlighted by Blue-Alcian stain. Small cystic spaces are also observed, even if these are more characteristic of low grade MEC (Blue-Alcian, 400×). **(C)** Cytologically, in the bronchial brushing of the same patient, atypical cells were recognized as intermediate cells after slide revision. These cells have a high nuclear/cytoplasmic ratio (Papanicolaou staining, 400×). **(D)** Squamous cells were also identified during revision of the slides, demonstrating atypical nuclei and more abundant cytoplasm. The inset shows cells with keratinizing cytoplasm (Papanicolaou staining, 400×). **(E**,**F)** Admixed intermediate cells (arrow) and mucin-secreting cells (asterisk); the abundance of mucin-secreting cells was the basis for diagnosis of adenocarcinoma on cytology **(E)** (Papanicolaou staining, 400×) **(F)** (Papanicolaou staining, 600×).

Mucoepidermoid carcinoma is the most common type of primary PSGT and it accounts for only 0.1–0.2% of all lung cancers (2, 28). In the majority of cases, it develops as an endobronchial lesion located in the central airways, namely trachea, carina, and main stem bronchi; less than 6% of patients present with a peripheral lung nodule (3, 28, 29). Prognosis of pulmonary MEC is significantly better than that of non-small cell lung cancer (NSCLC) and small cell lung cancer (SCLC). Five-year survival of these three entities is 88, 21 and <5%, respectively (30). When they are divided into high-grade and low-grade tumors, bronchial MEC show a 5-year survival of 31 and 80%, respectively (31). The cytological features of LRT MEC, which can be diagnosed by FNA, BB, BA, BW, and BAL, overlap those of their salivary gland counterpart. Three cell types should be identified from MEC histology: mucin-secreting, squamous, and intermediate cells, which can be organized in different architectural patterns (32). Low-grade tumors show cystic zones consisting of cytologically bland mucin-secreting cells and solid areas composed of squamous or intermediate cells. Mitoses and necrosis are rare. High-grade tumors mainly consist of atypical squamous and intermediate cells, accompanied by variable numbers of mucin-secreting cells; necrosis; and mitoses are frequent (Figures 1A,B) (3). On cytological specimens, various combinations of mucin-producing, squamous, and intermediate cells have been observed according to tumor grade, with the characteristic admixture of all three cell types being helpful for recognition of this entity (Figures 1C–F) (9, 14, 25): typical non-keratinized squamous cells show round nuclei and moderate cytoplasm; mucinous cells are variable in shape, have small uniform nuclei and prominent nucleoli, and may contain a single vacuole that displaces the nucleus; intermediate cells have well-defined homogeneous cytoplasm and small round nuclei with small nucleoli (25). Published cytological literature concerning primary pulmonary MEC shows that only Tao and Robertson and Brooks et al. have reported the presence of three distinct cell types (9, 14); all of the other authors described at best only two different cellular populations (Table 3) (11, 16, 18, 25). Other features encountered on MEC histology, such as the presence of intranuclear inclusions and clear cell change, have been occasionally described on cytology (18).

**Table 3:** **Cytomorphological features of primary pulmonary mucoepidermoid carcinoma (MEC) reported in the literature**.

Reference	Architecture	Background	Cell shape	Cytoplasm	Nuclei	Chromatin	Nucleoli
Tao and Robertson (9)	Tissue fragments with connective tissue core	ND	Spindle cells	Scanty	Ovoid	Finely granular, evenly distributed	Conspicuous in some cells
			Epidermoid cells	Apparent but not abundant	Round	Finely granular, evenly distributed	Conspicuous, prominent
			Mucus-secreting cells	Containing a large mucous vacuole	Round	ND	ND

Nguyen (11)	Single cells or small aggregates	Basophilic mucus-like material	Squamous cells (highly atypical)	ND	Large	ND	Prominent
			Mucus-secreting cells	Abundant, vacuolated	Small, vesicular	ND	ND

Brooks and Baandrup (14)	Small tissue fragments with papillary projections	ND	Polygonal cells	ND	Round or ovoid	Finely dispersed	Not prominent
	Occasional groups with fibrovascular core		Mucinous cells	Foamy, clear	ND	ND	ND
			Squamous cells	Abundant, dark blue, hyaline	Round, central	ND	ND

Segletes et al. (16)	ND	Clean	Glandular cells	Delicate	Eccentrical	ND	ND
			Squamoid/intermediate cells	Dense	Central	ND	ND

Romagosa et al. (18)	Cells either grouped in irregular aggregates or singly dispersed in mucin	Slightly mucinous	Epidermoid cells (with clear cell change)	Wide, loose, poorly defined	Round, intranuclear inclusions	Finely granular	ND
			Mucus-secreting cells	ND	ND	ND	ND

Dyhdalo and Chen (25)	Tight clusters	Extracellular mucus material	Small, bland cells	ND	Central, round, uniform	ND	Small
			Glandular cells	Vacuoles with mucin	ND	ND	ND

*ND, not described*.

**Figure 2:**
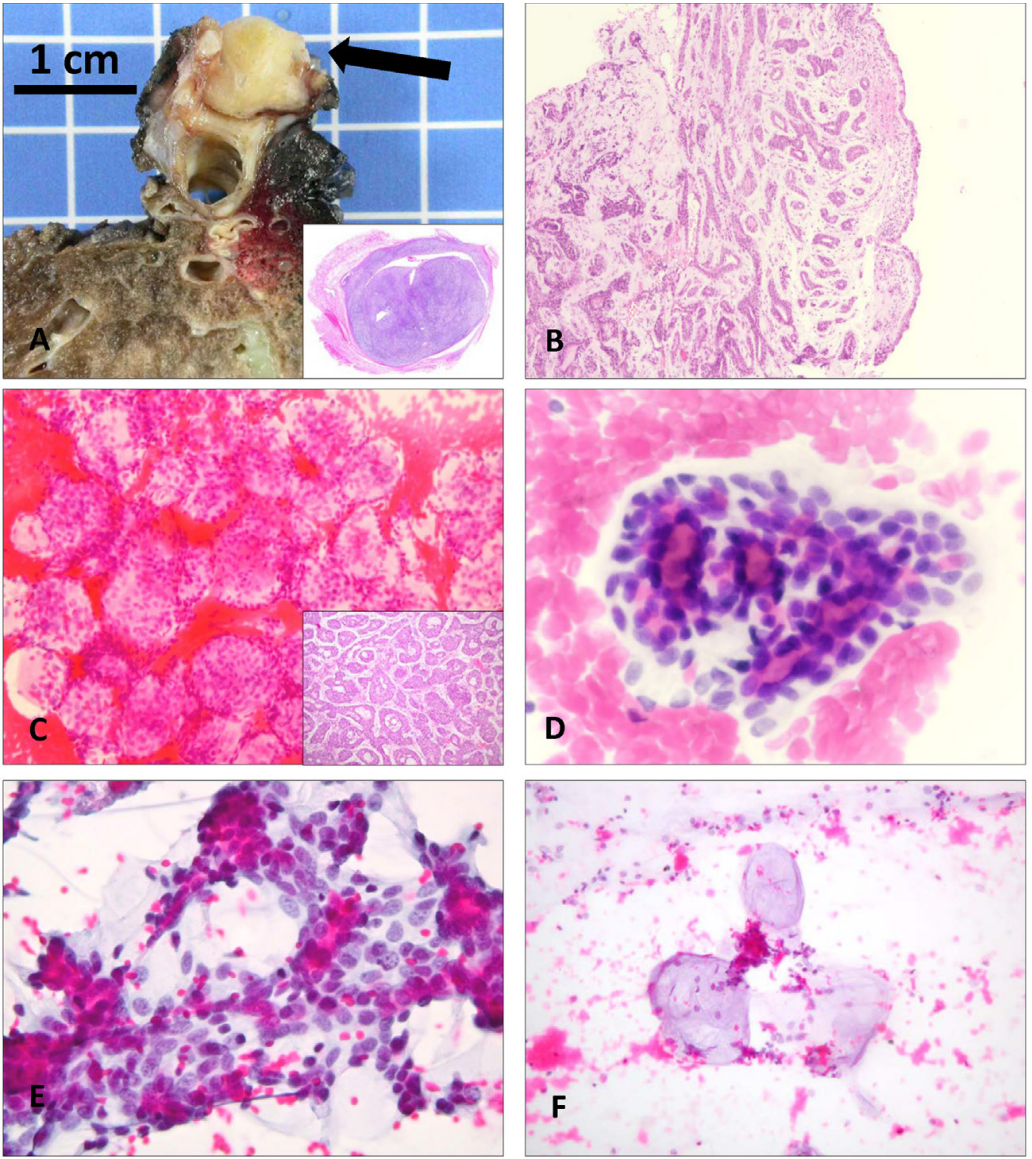
**Macroscopic, histological, and cytological aspects of primary adenoid cystic carcinomas (AdCC) of the lower respiratory tract**. **(A)** Macroscopic presentation of the 2.5-cm lesion in the distal carina of case 1. Inset shows the almost complete obliteration of the lumen of the bronchus (Hematoxylin and Eosin, scan of the slide). **(B)** Bronchial biopsy of case 4 demonstrates the typical cribriform and tubular pattern of AdCC (Hematoxylin and Eosin, 20×). **(C)** Ovoid structures constituted by monotonous cells surrounding a central lumen (“ball-like” clusters) that were considered as metastatic ductal breast carcinoma cells in case 2 (Papanicolaou staining, 200×). Inset shows the tubular architecture of the same case, exactly reflecting the cytological findings, which were correctly interpreted as primary AdCC on histology (Hematoxylin and Eosin, 20×). **(D)** Tubular structures comprised of repetitive medium-sized cells with scant cytoplasm and hyperchromatic nuclei containing a finely granular chromatin, containing a central core of homogeneous material. Note the inner layer of ductal cells and outer layer of myoepithelial cells (Papanicolaou staining, 400×). **(E)** The same cell types with scant pale-staining cytoplasm are arranged around hyaline globules in close proximity to each other (Papanicolaou staining, 400×). **(F)** Occasionally, the hyaline matrix is easily detected and is deprived of cells (Papanicolaou staining, 400×).

Adenoid cystic carcinoma also generally arises as an endobronchial tumor in central airways (Figures 2A,B); only sporadically is it reported in a peripheral lung location (4). Primary pulmonary AdCC is composed of two main cell types, ductal and modified myoepithelial cells, and can present three main architectural patterns, in keeping with salivary AdCC: cribriform, tubular, and solid (1, 4). Cytological findings include cohesive clusters of repetitive medium-sized cells, with scant cytoplasm and uniform, small, hyperchromatic nuclei containing a finely granular, evenly distributed chromatin (Figures 2C–F). Tumor cells are often arranged around a central core of homogeneous myxoid material, or form three-dimensional, “ball-like” clusters (Table 4) (10–13, 15, 19–22, 24, 26, 33). All of these features that recapitulate the histopathology of AdCC are helpful in correctly orienting the cytological diagnosis of this neoplasm. Sometimes, isolated hyaline globules can be observed (7, 20, 24, 26); singly dispersed cells are present on some smears (11, 26). The basement membrane material, forming globules that have a light blue appearance on PAP stain and bright magenta on MGG stain, is the characteristic feature of AdCC; diagnostic difficulties arise when they are not present on cytological material, as the pattern could mimic carcinoid tumor, SCLC, NSCLC, and reserve cell hyperplasia (19).

**Table 4:** **Cytomorphological features of primary pulmonary adenoid cystic carcinoma (AdCC) reported in the literature**.

Reference	Architecture	Background	Cell shape	Cytoplasm	Nuclei	Chromatin	Nucleoli
Lozowski et al. (10)	Cyst-like structures filled with dense, pink-staining, amorphous material (rarely)	Pinkish-staining, mucous, granular background	ND	ND	Uniform, small, ovoid	Finely granular, evenly distributed	ND

Buchanan et al. (12)	Cohesive clusters of cells with central cystic spaces filled with amorphous, hyaline material	ND	ND	Minimal	Uniform, small, ovoid	Finely granular, bland	ND
	Three-dimensional, ball-like formations						

Nguyen (11)	Single and clustered tumor cells	ND	Cuboidal	Scanty	Round, hyperchromatic	ND	ND
	Gland-like spaces filled with pinkish mucus-like material						
Gupta and McHutchison (13)	Cohesive three-dimensional clusters of cells; cystic spaces containing cyanophilic amorphous material	ND	Uniform	Minimal	Uniform, small, ovoid	Finely granular	ND

Radhika et al. (15)	Mucoid globules surrounded by malignant cells	ND	Cylindroid/tubular	Scanty	Hyperchromatic	ND	ND
	Solid clusters of cells						

Segletes et al. (16)	Tightly cohesive aggregates	Clean	Small, uniform	Scant, delicate, non-vacuolated	Ovoid, high nuclear/cytoplasmic ration	Finely granular, evenly distributed, darkly stained	ND
	Clusters of cells including central acellular spheres of dense, homogeneous material						
Özkara and Turan (23)	Three-dimensional clusters of neoplastic basaloid cells associated with hyaline basement membrane material	Bloody	Homogeneous, small	Modest, eosinophilic	Small, hyperchromatic	ND	ND

Qiu et al. (19)	Three-dimensional clusters of neoplastic basaloid cells associated with hyaline material forming cylinders or spheres	ND	ND	ND	ND	ND	ND
	Aggregates of neoplastic basaloid cells with scanty or no amorphous material						

Florentine et al. (20)	Scattered sheets and ball-like clusters of tumor cells	ND	Small, basaloid	Scanty	Round	ND	ND
	Hyaline globules at times surrounded by neoplastic cells						

Chuah et al. (21)	Solid sheets and gland-like spaces associated with mucoid material	ND	Monomorphic	ND	ND	ND	ND
	Tight, branching clusters with tubular appearance						

Daneshbod et al. (22)	Cell clusters associated with myxoid, hyaline material	ND	Dimorphic appearance of tumor cells	ND	ND	ND	ND

Chon et al. (24)	Tight clusters, globules of acellular mucoid material	ND	Monomorphic, basaloid	ND	Round to oval	Fine granular	Indistinct

Bhalara et al. (26)	Poorly cohesive clusters and complex sheets	ND	ND	Scanty	Monomorphic, bland, hyperchromatic	ND	ND
	Homogeneous hyaline globules						
	Singly dispersed cells						

Kim et al. (7)	Organoid clusters	ND	Small, uniform, hyperchromatic	Granular	ND	ND	Distinct
	Sheet formation						
	Hyaline globules						

*ND, not described*.

Retrospectively, a correct preoperative diagnosis of all AdCC could have been rendered, because characteristic tridimensional clusters and hyaline globules were present on the smears; the hyaline globules were confused with a metastatic breast carcinoma in the original diagnosis of one case and considered suspicious for carcinoma, NOS, in the other. Considering MEC, only one additional case could have been identified. The cytological diagnosis of MEC was possible since all the three diagnostic cellular components were present with features of malignancy (i.e., squamous, glandular, and intermediate cells). When looking carefully at the smears, it was possible to identify aggregates of medium-sized cells that were bigger than basal and reserve bronchial cells. In the original cytological diagnosis, these intermediate cells were considered as suspicious for a carcinoma, NOS. Of note, in this case, an Alcian Blue staining was performed to identify glandular neoplastic cells, but only normal bronchial mucous cells were seen. Retrospective analysis revealed that the cells defined as “normal bronchial cells,” which stained positive for Blue-Alcian, were actually atypical. This allowed the retrospective diagnosis of MEC. In the remaining cases, the criteria for MEC were not fulfilled and only a poorly differentiated carcinoma could be diagnosed.

Immunocytochemistry is of limited value in diagnosing PSGT of the LRT. If these histological subsubtypes are not considered, only traditional markers of NSCLC subtyping are used. While epithelial cells of MEC and ductal cells of AdCC are positive for common epithelial markers (such as CK7 and CK 5/6) and p63 and p40 are expressed in all the intermediate and squamous cell component of MEC, myoepithelial cells of AdCC are usually positive for smooth muscle actin, vimentin, myosin, S-100, and for p63. Thus, CK7, CK5/6, p63, and p40 are potentially misleading markers as they are also part of the immunocyto-/histochemical panel used to classify lung carcinomas. Their positivity would lead to a diagnosis of primary lung squamous cell carcinoma, rather than pointing to the presence of a squamous cell component in MEC or to the myoepithelial differentiation typical of AdCC (34, 35).

Besides these more common entities, other rarer PSGT of the LRT include acinic cell carcinoma, PA with its malignant counterpart carcinoma ex PA, myoepithelioma and myoepithelial carcinoma, mucous gland adenoma, and oncocytoma (1, 33, 36, 37). No cytological description of such lesions in the LRT has been reported. Recently, a case of a primary pulmonary mucin-rich variant of salivary duct carcinoma with preoperative cytology was published: BAL revealed cytologic atypia, and the right upper lobe bronchial brushing was positive for carcinoma. However, ICC was not performed due to the paucity of diagnostic material and a conclusive diagnosis was not reached on cytological material (38). MASC, a rare salivary gland tumor first described in 2010, has never been described as a primary lung neoplasm (39). While reviewing the cytological and histological slides for our study, we paid particular attention to the identification of features that could point to a diagnosis of MASC, which we did not observe. No features resembling acinic cell carcinomas, that could have warranted (on cytological as well as on histological material) an immunocytochemical analysis for mammaglobin or FISH/molecular analysis for ETV6-NTRK3 translocation or ETV6 break, were seen (40, 41). ETV6-NTRK3 translocation or ETV6 breaks are present in up to 80 and 99% of MASC cases, respectively, and are quite specific for this entity (8).

In recent years, in addition to this molecular feature characteristic of MASC, other diagnostic molecular signatures have been described for salivary gland tumors, even the ones developing in the LRT, and some with a high prevalence and discrete specificity (8, 34, 35, 42). With respect to MEC, specific translocations involving the CRTC1 gene and MAML2 or CRTC3 and MAML2 have been described, with frequencies up to 80 and 6% respectively (8, 43, 44). AdCC is characterized by a specific translocation, namely *MYB/NFIB*, present in up 90% of cases (8, 45). This translocation results in MYB protein overexpression that can be detected using IHC (46, 47). This test can be particularly useful to confirm the diagnosis of AdCC, especially when combined with c-KIT (CD117) positivity, and can be applied on cytological smears. However, immunohistochemical staining for CD117 cannot be used alone in differential diagnosis of salivary gland neoplasms, because AdCC, PA, polymorphous low-grade adenocarcinoma, and monomorphic adenoma have all been found to be positive, to differing degrees, for CD117. The use of a panel of immunomarkers including MYB, CD117, and the zinc finger protein PLAG1 (PA gene 1), quite specific for PA, is more judicious and very effective (46–48). A search for the *EGFR* mutations was performed on the resected specimens of only one of our AdCC cases, which gave a negative result. Usually these tumors do not have *EGFR* mutations (49), although one case of AdCC with *EGFR* mutations has recently been reported (50). In our series (MEC and AdCC), molecular techniques could have been applied on cytological material in the case of diagnostic doubt, in order to detect these specific molecular alterations. However, apart the search for the *EGFR* mutation that has been done for therapeutic reasons, no molecular test was originally performed, not even for more recent cases. This supports the hypothesis that a diagnosis of primary PSGT was not considered.

## Conclusion

An awareness of the possibility of encountering primary PSGT in the cytological specimens of patients investigated for LRT masses is fundamental to establishing a correct diagnosis. This is particularly relevant for AdCC, as all cases reported in literature showed characteristic cytological features that could have allowed a correct preoperatory diagnosis. As far as MEC is concerned, its preoperative diagnosis is more difficult, as the three different cellular components (i.e., squamous, intermediate, and mucin-secreting cells) were not always reported to be present on cytology specimens. In cases that raise suspicion of AdCC or MEC, additional immunohistochemical (MYB, c-kit) or molecular techniques (e.g., FISH) could be applied to cytological smears to refine the diagnosis.

## Ethics Statement

The study protocol was approved by the regional ethical commission on research and human beings (CER-VD, 2016-00224). Informed consent was not necessary according to the art. 34 of the Federal Act on Research involving Human Beings (Human Research Act, HRA); data concerning study participants were anonymized.

## Author Contributions

MB conceived the idea of the project. MB, MV, GG, and MP contributed to identification of cases and data curation. CS and MB prepared the manuscript. MV, SLR, IL, MP, and MB reviewed the manuscript. All authors edited the manuscript before its submission.

## Conflict of Interest Statement

The authors declare that the research was conducted in the absence of any commercial or financial relationships that could be construed as a potential conflict of interest.
